# Q‑Learning-Based Multivariate Nonlinear Model Predictive Controller: Experimental Validation on Batch Reactor for Temperature Trajectory Tracking

**DOI:** 10.1021/acsomega.5c03482

**Published:** 2025-06-26

**Authors:** Abhiram Varma Vegesna, Muralikrishna Shamaiah Narayanarao, Kishore Bhamidipati, Thirunavukkarasu Indiran

**Affiliations:** † Department of Computer Science and Engineering, Manipal Institute of Technology, 125853 Manipal Academy of Higher Education, Manipal, Karnataka 576 104, India; ‡ Department of Instrumentation and Control Engineering, Manipal Institute of Technology, 76793 Manipal Academy of Higher Education, Manipal, Karnataka 576 104, India

## Abstract

This study introduces a Q-learning-based nonlinear model predictive control (QL-NMPC) framework for temperature control in batch reactors. A reinforcement learning agent is trained in simulation to learn optimal control strategies using coolant flow rate and heater current as inputs. The resulting policy, represented as a Q-table, is implemented in real time on a physical reactor setup using the NVIDIA Jetson Orin platform. The proposed QL-NMPC framework employs a value iteration-based Q-learning algorithm, enabling model-free policy optimization without explicit policy evaluation steps, and demonstrates effective temperature tracking while highlighting the potential of reinforcement learning for controlling nonlinear batch processes without relying on system identification.

## Graphical abstract

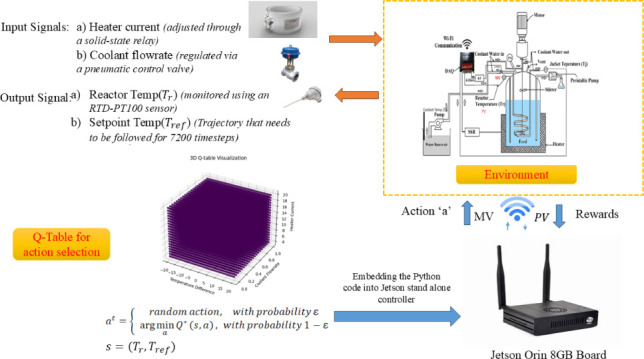

## 1 Introduction

Batch reactors are widely employed in the chemical and pharmaceutical industries due to their flexibility in handling various reaction schemes and product specifications. Their ability to operate under time-varying conditions makes them ideal for producing specialized, high-value chemicals. Before being scaled up to continuous production systems, batch reactors are often the preferred choice in research and development settings for studying reaction kinetics and optimizing process conditions.

Despite their versatility, batch reactors present significant operational challenges. Their inherently nonlinear and time-dependent dynamics complicate the development of accurate models and reliable control strategies. Traditional control methods, often based on single-variable models, fall short in handling multivariable interactions and process constraints, leading to issues such as excessive energy usage, inconsistent product quality, and poor batch-to-batch repeatability.^1,2,5^ Moreover, precise control of the temperature and reactant dosing is critical to avoid undesired side reactions and ensure safe, efficient operation.

To address these control challenges in batch reactors, this work explores a data-driven, model-free control strategy based on reinforcement learning. Among various reinforcement learning techniques, Q-learning is a widely used algorithm that enables agents to learn optimal actions by maximizing cumulative rewards through interactions with an environment, without requiring an explicit model of the system. It uses a value-based approach, where the agent builds a Q-table that maps state-action pairs to the expected future rewards.

Leveraging this method, a Q-learning-based Nonlinear Model Predictive Control (QL-NMPC) framework is developed and trained in a simulated environment, where the agent learns to regulate temperature through interaction with a physics-based reactor model. Starting with a Single-Input Single-Output (SISO) configuration using coolant flow rate, the control strategy is extended to a Multi-Input Single-Output (MISO) setup by introducing heater current. A reward function is carefully designed to penalize temperature deviations and promote smooth control behavior. Once trained, the Q-table is deployed to a real-time hardware platform for closed-loop control. This methodology offers a scalable, simulation-driven solution for managing complex dynamics in chemical process control.

To present a brief overview of the recent works carried out in batch reactor, Q-learning, NMPC, and QL-based deep learning recent literature is consolidated to facilitate a quick understanding of blending Q-learning with nonlinear model predictive controllers.

Prajwal et al.^1^ developed and experimentally validated a nonlinear model-based control (NMBC) strategy for batch polymerization reactors. This method, which integrates an unscented Kalman filter (UKF), demonstrated an impressive improvement in trajectory tracking while achieving reduced computational costs compared with traditional nonlinear model predictive control (NMPC). The UKF helped to account for nonlinearity and state estimation uncertainties in real-time, making the strategy more efficient and adaptable for real-world applications. This work highlighted the practical feasibility of implementing NMBC for trajectory tracking in complex chemical processes, emphasizing its potential for industrial applications where computational efficiency and accurate control are critical.

Aishwarya et al.^2^ proposed a hybrid convolutional neural network (CNN)-long short-term memory (LSTM) model within an NMPC framework to control batch reactor temperatures. The hybrid model effectively combined CNN’s spatial feature extraction capability with LSTM’s ability to capture temporal dependencies, resulting in highly accurate predictions. By incorporating a sigmoidal heuristic, the authors significantly improved the system’s efficiency, leading to a substantial reduction in mean squared error. This study demonstrated the power of deep learning in capturing both spatial and temporal dynamics in industrial control, suggesting that such hybrid models could offer better adaptability and robustness for complex dynamic systems, such as batch reactors.

Arockiaraj et al.^3^ employed a multi-kernel support vector machine (SVM) approach for fault detection in batch reactors. Their methodology, which explored various kernel functions such as the radial basis function (RBF), achieved over 98% accuracy in fault detection. The study highlighted the significance of appropriate kernel selection and feature scaling to handle the complex nonlinear patterns often present in industrial processes. The work also underscored the importance of hyperparameter tuning in machine learning models to ensure robustness in fault detection, suggesting that SVMs can be promising tools for real-time monitoring and fault diagnosis in chemical production systems.

Zhang et al.^4^ proposed a Q-learning-based model predictive control (MPC) strategy for nonlinear continuous-time systems using actor-critic neural networks and Lyapunov-based techniques. Their approach aimed to reduce online optimization, which is a common bottleneck in traditional MPC frameworks, while maintaining system stability through the Lyapunov methods. While the method was effective in reducing computational effort, it required a rich data set for training, which could be a challenge in some real-world applications where obtaining such data is difficult. Moreover, the robustness of the system to model uncertainties was somewhat limited, indicating that further research into improving its adaptability to varying conditions would be beneficial.

Bagla et al.^5^ introduced output feedback and adaptive economic NMPC (ENMPC) frameworks to address model mismatch issues in real-time optimization (RTO)-based MPC systems. The adaptive ENMPC variant performed exceptionally well by incorporating real-time parameter estimation to adjust to plant-model mismatches. Despite its promising performance, the approach faces challenges related to computational load, as it requires frequent reestimation of parameters, which may not be feasible in time-sensitive operations. Moreover, both approaches are heavily reliant on good initial estimates, which can limit their applicability in systems with poor prior knowledge.

Jang et al.^6^ provided a thorough classification of Q-learning algorithms, distinguishing between single-agent and multiagent methods. They reviewed the use of Q-learning in robotics and control systems, providing insights into its limitations, such as the potential for overestimation of Q-values and scaling issues in large systems. The paper suggested that while Q-learning has shown great promise, there is a need for more robust and scalable designs, especially when it comes to addressing the challenges posed by large state and action spaces and real-time learning.

Kordabad and Gros^7^ proposed a method for verifying dissipativity and computing storage functions in economic NMPC using undiscounted Q-learning. Their approach, which transforms the economic cost function into a tracking form, was successful in optimizing the economic performance. However, the method relies heavily on expressive approximators, which can become problematic in nonpolynomial systems where approximations may not capture the true dynamics of the system. This work highlighted the trade-off between model accuracy and computational feasibility, suggesting the need for more flexible approximation methods to handle complex systems.

Adhau et al.^8^ developed a fast RL-based MPC method using nonlinear programming (NLP) sensitivities to approximate the Q-function, significantly reducing the computational burden by avoiding repeated optimizations. While the method was effective for systems with relatively simple dynamics, its accuracy decreased in the presence of highly nonlinear systems, limiting its applicability in more complex industrial processes. This research emphasized the potential for combining RL with optimization techniques to achieve real-time performance but also pointed out the challenges of maintaining high accuracy in nonlinear environments.

Konar et al.^9^ proposed an improved Q-learning algorithm (IQL) for mobile robots, which locks Q-values based on known distances to improve convergence and reduce path complexity. This enhancement allowed the algorithm to efficiently navigate static environments. However, the IQL method assumes a static environment, which poses challenges when dealing with dynamic, real-time changes in the environment. Future work could focus on integrating real-time sensory data to improve adaptability and robustness in dynamic scenarios, making the algorithm more practical for autonomous navigation in unpredictable settings.

Lei and Liu^10^ presented a deep Q-network (DQN)-based strategy for robot navigation in corridor environments. The approach combined supervised feature extraction with reinforcement learning to ensure that robots avoided obstacles while navigating. However, the reliance on pretrained feature extraction limited the algorithm’s ability to generalize to unfamiliar environments, reducing its flexibility. The study highlighted the importance of balancing the use of pretrained features with the ability to adapt to new, unseen environments, suggesting that further advancements in feature extraction and transfer learning could enhance the system’s adaptability.

Wei et al.^11^ introduced a deterministic Q-learning method with a relaxed convergence analysis that updates all Q-values per iteration, ensuring guaranteed convergence. While this approach provides certainty about the system’s eventual stabilization, it also comes with the downside of increased computational complexity, especially when dealing with large state-action spaces. This method is best suited for deterministic systems, but its scalability to more complex stochastic systems remains a challenge, necessitating the development of more efficient algorithms for broader applications.

Idris and Engell^12^ proposed an economic NMPC strategy for catalytic distillation processes, achieving superior economic performance through advanced optimization techniques. Despite its success, the method faced challenges due to model sensitivity and the complexity of the nonlinear optimization involved. This research suggested that while the economic NMPC approach is effective for improving process economics, the sensitivity to model parameters and the difficulty of solving complex nonlinear problems in real-time remain significant hurdles that need to be addressed through more efficient solvers and robust modeling techniques.

Lucia et al.^13^ introduced a multistage NMPC framework designed to optimize economic performance under uncertainty. While the method was effective, it was constrained by the high computational load required for multistage optimization, which limited its practical applicability in real-time industrial scenarios. The study highlighted the trade-off between achieving robust optimization and maintaining feasibility in computationally demanding tasks, recommending that future research focus on reducing computational requirements while maintaining the quality of economic optimization in uncertain environments.

Wolf and Marquardt^14^ reviewed fast NMPC schemes that focus on suboptimal control and sensitivity updates, aiming to reduce delays in economic NMPC systems. While the methods presented promising results in improving the speed of optimization, they struggled with changes in active sets, which can lead to instability in the optimization process. The authors suggested that exploring parallel computing and adaptive techniques could provide potential solutions to mitigate these challenges and enhance the efficiency and stability of fast NMPC schemes.

Alhazmi et al.^15^ proposed an RL-based economic model predictive control (EMPC) system for chemical reactors, incorporating online model updates to address plant-model mismatch. While the framework showed promise for autonomous control, it was hindered by high computational demands, particularly when updating models in real time. This research underscored the need for more computationally efficient RL algorithms capable of handling model uncertainties in complex chemical processes without compromising performance.

Zanon et al.^16^ embedded real-time iteration NMPC (RTI-NMPC) into reinforcement learning as a differentiable function approximator. This hybrid approach enabled fast control actions but may compromise long-term optimality due to approximations of system dynamics. The success of this method is highly dependent on the accuracy of the approximations, which can be difficult to achieve in complex systems. Future work could focus on improving the precision of function approximations and exploring trade-offs between speed and long-term optimality in real-time applications.

Chen et al.^17^ introduced fidelity-based probabilistic Q-learning (FPQL) for quantum system control. The method uses fidelity as a guide for exploration, improving learning efficiency by focusing on areas of high probability. However, the approach is computationally intensive and lacks scalability, which limits its applicability to large-scale quantum systems. This work suggests that while FPQL has potential for quantum control, further research is needed to enhance its scalability and reduce its computational demands.

Matignon et al.^18^ developed Hysteretic Q-learning for decentralized learning in cooperative multiagent systems. The algorithm used dual learning rates to address uncertainty and improve coordination among agents. While the method showed improved performance in multiagent environments, its effectiveness depends heavily on the careful tuning of learning rates, which can be challenging. This research highlights the need for adaptive learning rates and more flexible approaches to handle uncertainties in cooperative systems.

Luo et al.^19^ proposed critic-only Q-learning (CoQL), which estimates the Q-function using a single neural network without the need for solving Hamilton–Jacobi–Bellman (HJB) equations. This approach avoids reliance on model-based solutions but requires extensive offline data and precise parameter tuning. The study indicated that while CoQL could simplify the learning process, it still faces challenges related to data quality and the need for large amounts of offline training data.

Wang and Zhan^20^ reviewed various reinforcement learning algorithms, including SARSA and Q-learning, and their applications in control systems and robotics. They identified key challenges such as slow convergence and issues with generalization, particularly in large, complex systems. The review recommended exploring multiagent frameworks and improved exploration strategies to address these issues and enhance the scalability and robustness of RL algorithms in real-time control applications.

Huang and Biegler^21^ integrated electricity price forecasting using ARIMA models into NMPC for energy-intensive processes. By adjustment of operational strategies based on forecasted electricity prices, the controller achieved significant cost savings. However, the approach’s effectiveness is limited by the accuracy of the price forecasts, which can be difficult to predict with high certainty. This work highlighted the importance of accurate forecasting in economic NMPC and suggested that further research into more robust forecasting methods could improve the system’s overall performance.

Liu et al.^22^ proposed a Q-learning-based routing protocol (QMR) for Unmanned Aerial Vehicle (UAV) networks, optimizing energy consumption and delay in real-time scenarios. While the protocol showed promising results in improving network performance, it faced difficulties in maintaining stable neighbor relationships in highly dynamic environments. Future work could explore adaptive strategies that handle dynamic changes in UAV network topology, improving the protocol’s performance in real-time applications.

Griffith et al.^23^ developed a robust economic NMPC framework that maintained stability for nondissipative systems through a stabilizing constraint. While the framework was highly flexible, it encountered significant computational challenges when applied to large-scale systems. The study suggested that future research should focus on improving the scalability of such systems through parallel computing techniques and more efficient optimization algorithms to overcome computational limitations in large-scale applications.

## 2 Proposed Approach

### 2.1 Batch Reactor Dynamics

Batch reactors exhibit strongly nonlinear behavior and tend to consume significant energy during operation. Numerous studies have explored trajectory tracking in simulation environments, while only a few have extended their work to include experimental validation. Most of these experimental studies have relied solely on manipulating the coolant flow rate as the primary control input, while keeping the heater current fixed. This approach, however, often results in increased thermal energy consumption and excessive coolant demand to ensure that the reactor temperature follows the desired reference trajectory.

In contrast, this work introduces a multivariable nonlinear model predictive control (NMPC) strategy aimed at improving both the energy efficiency and tracking performance. The controller is designed to follow a predefined temperature trajectory over time, which is crucial for achieving desired reaction yields and avoiding undesirable side reactions (see Figure 2 for the reference trajectory used in this study).

Figure 1 illustrates the layout of the laboratory-scale batch reactor, which is equipped with two manipulated variables (MVs): coolant flow rate (Fc) and heater current (Hc). The reactor temperature (PV1) is monitored using an RTD-PT100 sensor. A cyber-physical control loop is established through socket-based communication between a Wi-Fi-enabled DAQ module (ACE-2007) and an NVIDIA Jetson Orin board, where the control algorithm is executed in real time.

**Figure 1:**
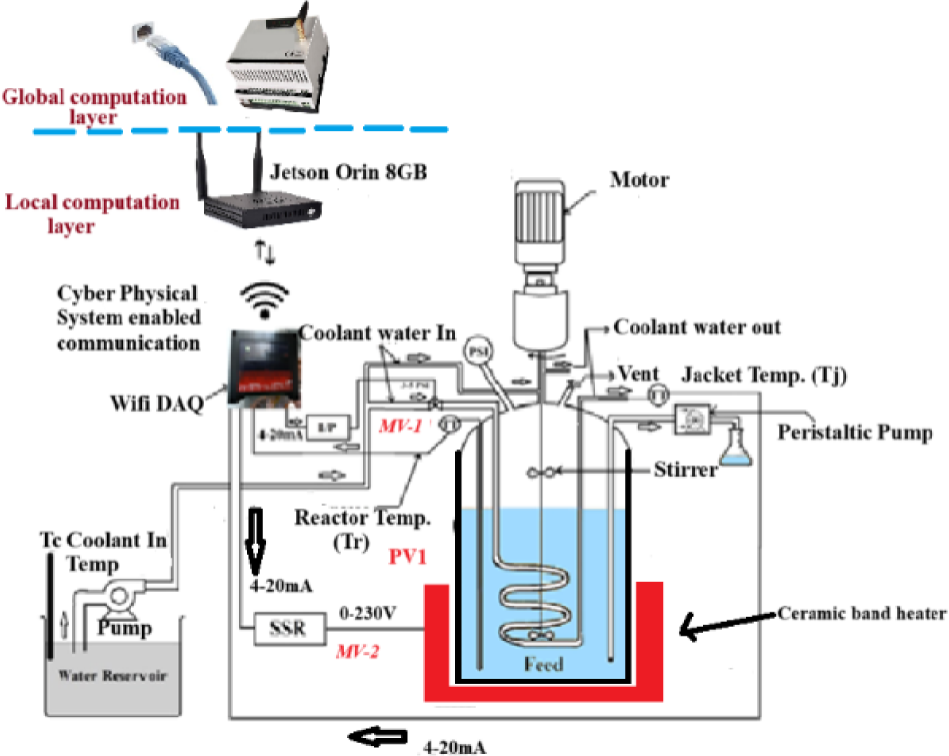
Schematic diagram of lab-scale reactor with multi input and single output structure with Jetson Orin board enabled with cyber physical system available in Machine Learning for Advanced Process Control Lab, MIT, Manipal.

The coolant flow is regulated via a pneumatic control valve (MV1), and the heater current is adjusted through a solid-state relay (MV2), which operates on a 4–20 mA control signal. The RTD signals corresponding to the reactor and jacket temperatures are calibrated for a 0–100 °C operating range. The heating mechanism employs a ceramic band heater mounted around the reactor with a small air gap, facilitating indirect heat transfer through radiation.

At the end of the batch reactor modeling, an optimal trajectory has been designed for the monomer-to-polymer conversion for a fixed batch time; e.g., in this case, it is 60 min. Usually, the batch reactor follows the golden batch time principle, which means that 60–65% of monomer conversion will occur within 50–60% of the desired temperature profile. Consequently, the profile exhibits a falling temperature trend toward the end of the desired temperature profile, leaving a minimum temperature for the remaining monomer to polymer. This trend is reflected in the reference trajectory shown in Figure 2. The formation of the optimal temperature profile varies depending on the different feeds used. Therefore, the required optimization technique must be applied. Additionally, online optimization of trajectory reformation can be performed based on the conversion rate.

**Figure 2:**
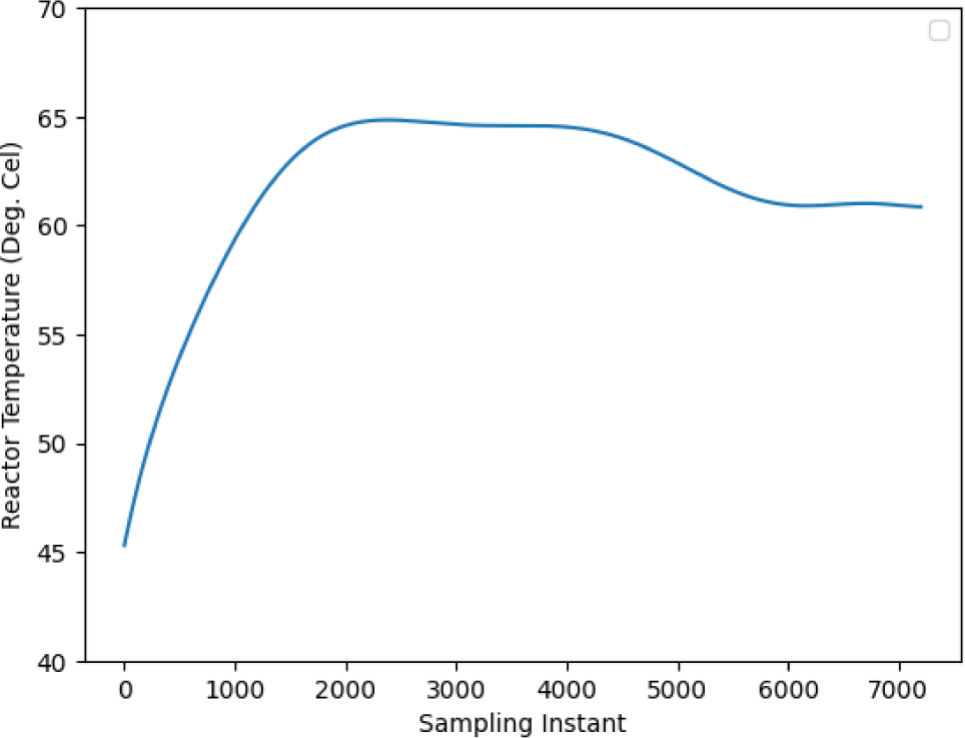
Trajectory defines the desired temperature profile to be followed throughout the reaction duration to ensure an optimal product yield and reaction efficiency.

### 2.2 SISO QL-NMPC Design for Simulation

This section outlines the design of a single-input single-output (SISO) Q-learning-based nonlinear model predictive control (QL-NMPC) strategy, where the reactor temperature (*T*_r_) is the controlled output, and the coolant flow rate (U1) is the sole manipulated input.

#### 2.2.1 Policy

A control policy π(s) is defined to determine the optimal coolant flow rate based on the current system state. An ε-greedy policy is adopted to encourage exploration during early learning and convergence to an optimal policy thereafter:

1 $$\pi \left(s\right)=\{\begin{array}{l} \mathrm{random action, with probability}\;\varepsilon \\ \arg\max Q^{*}\left(s,a\right),\mathrm{with probability}\;1-\varepsilon \end{array}$$

Here, s= (*T*_r_, *T*_ref_) represents the current state of the reactor in terms of actual and reference temperatures.

#### 2.2.2 Reward Function

The reward function is designed to penalize tracking error and encourage stable control behavior:

2 $$r\left(s,a\right)=-\left(Q_{c}{\left(T_{r}-T_{\mathrm{ref}}\right)}^{2}+R_{c}{\left(\Delta U\right)}^{2}\right)$$

where: *T*_r_: reactor temperature at time step *t*, *T*_setpoint_: set point temperature at time step t, ΔU: change in control input (coolant flow rate) compared to the previous step, *Q*_c_ > 0: weight for penalizing temperature tracking error, *R*_c_ > 0: weight for penalizing actuator variation.

This formulation ensures that both the temperature accuracy and actuator smoothness are taken into account. The negative sign is necessary because we aim to maximize the reward, which corresponds to minimizing the cost.

#### 2.2.3 Value Function

The value function V^π^(*s*) estimates the expected cumulative future reward over the next H steps under a given policy π:

3 $$V^{\pi }\left(s\right)=\overset{H}{\underset{t=0}{\sum }}\gamma^{t}r\left(s_{t},a_{t}\right)$$

The discount factor γ∈[0,1) determines the importance of future versus immediate rewards.

#### 2.2.4 Q-Table and Update Rule

A Q-table is maintained to store the expected long-term reward with a predictive horizon H for each state-action pair (s,a). The Q-learning update is performed using the following rule:

4 $$Q(s,a)\leftarrow {Q(s,a)+\alpha (R+\gamma }^{H}\max_{a^{\prime }}Q^{*}\left(s^{\prime },a^{\prime }\right)-Q\left(s,a\right))$$

where

•*Q*(*s*, *a*)→ current estimate of the value for taking action *a* in state *s*
•R → accumulated reward over H timesteps
•γ → discount factor, which determines the importance of future rewards
•max_*a*′_ *Q* ^*^(*s*′, *a*′)→ highest expected reward achievable from the next state (essentially the value function V­(s′) under the optimal policy)

#### 2.2.5 Optimal Control Action

The optimal coolant flow rate u* at each time step is derived as:

5 $$u^{*}=\arg{\mathrm{ma}x}_{a}Q^{*}\left(s,a\right)$$

Actions are discretized into finite bins over a permissible control range (0,1), and this discretization enables the implementation of Q-learning in a continuous action environment.

Figure 3 shows the simulation results of the Q-learning-based NMPC for the SISO configuration. Figure 4 shows the flowchart algorithm used for training the Q-learning table.

**Figure 3:**
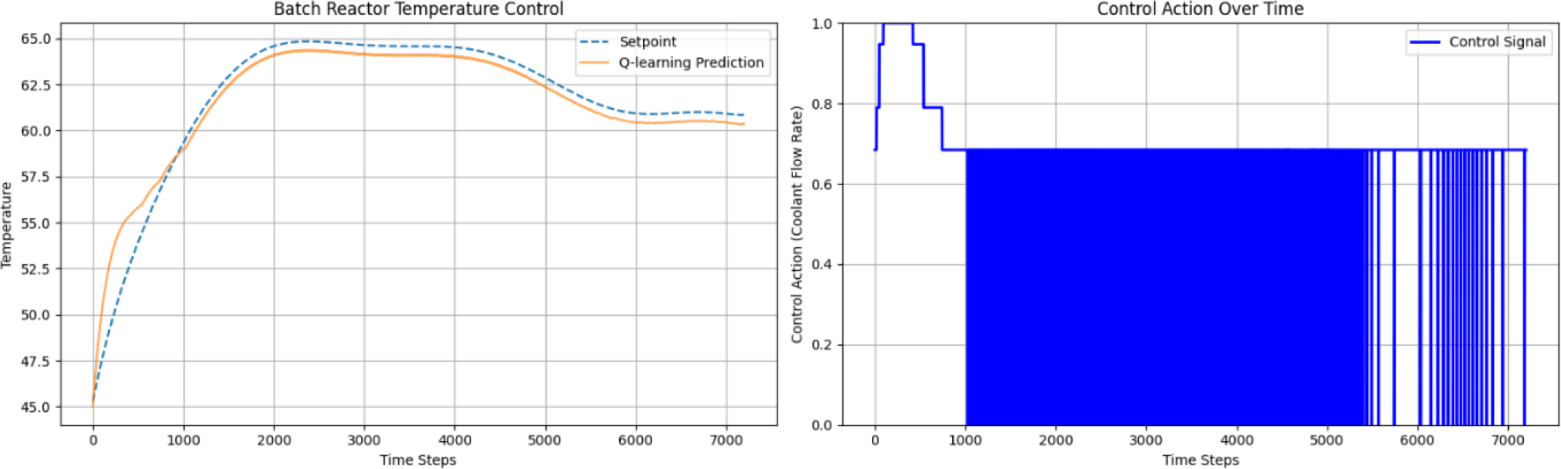
Simulation response of the temperature tracking and its control signal plot generated with the SISO structure of the batch reactor with a trained Q-table.

**Figure 4:**
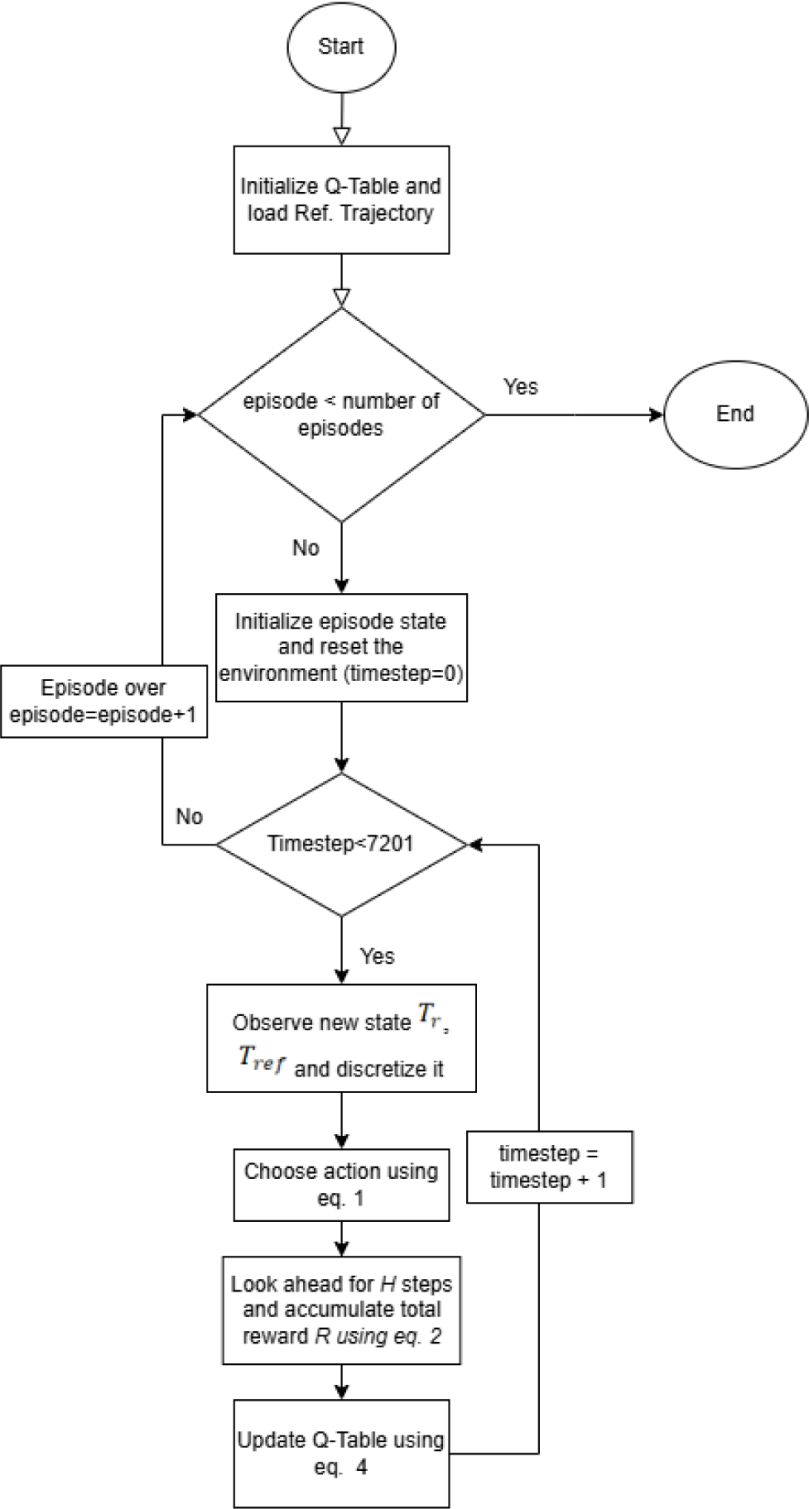
Flowchart of the Q-learning algorithm used for training.

### 2.3 MISO QL-NMPC Design for Simulation

This section details the design of a multiple-input single-output (MISO) Q-learning-based nonlinear model predictive control (QL-NMPC) strategy. In this framework, the reactor temperature (*T*_r_) is the sole controlled output, whereas two manipulated inputs—the coolant flow rate (U_1_) and the heater current (U_2_)—are applied to influence the process dynamics.

#### 2.3.1 Policy

A control policy π(s) is established to determine the optimal control actions based on the current state of the reactor. The policy is constructed using an ε-greedy approach. This approach initially encourages exploration of the action space to adequately sample the system dynamics and then gradually transitions toward exploiting the most promising control actions as learning converges. In the MISO setup, the state and action are defined as follows:

•State, *s*:

*s* = (*T*_r_, *T*_ref_)

where *T*_r_ is the reactor temperature at the current time step, and *T*_ref_ is the reference set point.

•Action, *a*:

a = (U_1_, U_2_)

where U_1_ corresponds to the coolant flow rate and U_2_ corresponds to the heater current.

#### 2.3.2 Reward Function

The reward function is designed to balance the competing requirements of accurate temperature tracking and smooth actuator operation. The function penalizes deviations of the reactor temperature from the set point as well as large variations in both control actions between time steps. It is expressed mathematically as

6 $$r\left(s,a\right)=-\left(Q_{c}{\left(T-T_{\mathrm{setpoint}}\right)}^{2}+R_{c1}{\left(\Delta U_{1}\right)}^{2}+R_{c2}{\left(\Delta U_{2}\right)}^{2}\right)$$

where:

*T*: Reactor temperature at time step *t*.

*T*_setpoint_: set point temperature at time step *t*.

Δ*U*_1_: change in coolant flow rate relative to the previous time step.

Δ*U*1: change in heater current was relative to the previous time step.

*Q*_c_ > 0: weighting factor for the state (temperature) error.

*R*_c1_ > 0, *R*_c2_ > 0: weighting factors penalize abrupt changes in the coolant flow rate and heater current, respectively.

The inclusion of the negative sign aligns the formulation with the objective of maximizing the reward, thereby implicitly minimizing the associated cost terms.

#### 2.3.3 Value Function

7 $$V^{\pi }\left(s\right)=\overset{H}{\underset{t=0}{\sum }}\gamma^{t}r\left(s_{t},a_{t}\right)$$

To assess the effectiveness of a particular policy, the value function *V* ^π^(s) is employed. This function estimates the expected cumulative reward over a predictive horizon *H* when following the control policy π.

Here, γ ∈ [0, 1) is the discount factor, which moderates the influence of future rewards relative to immediate rewards. A lower value of γ places greater emphasis on short-term performance, whereas values closer to 1 incorporate long-term benefits into the decision-making process.

#### 2.3.4 Q-Table and Learning Update

Central to the QL-NMPC strategy is the Q-table, which stores the estimated cumulative long-term reward for each state-action pair (s, a) over the predictive horizon *H*. The learning algorithm updates the Q-values based on observed rewards and projected future rewards using the following rule:

8 $$Q(s,a)\leftarrow {Q(s,a)+\alpha (R+\gamma }^{H}\max_{a^{\prime }}Q^{*}\left(s^{\prime },a^{\prime }\right)-Q\left(s,a\right))$$

where

*R* → accumulated reward over H timesteps.

γ^*H*^max_a′_Q^*^(*s*′, *a*′)→ discounted future value (signifies how valuable the next state is)

*s*′: the subsequent state after executing action *a*.

max_a′_Q^*^(*s*′, *a*′): represents the optimal future value estimation based on the next state *s*’.

This update rule ensures that the value estimates are progressively refined based on both immediate performance and future expectations.

#### 2.3.5 Control Action Selection

Unlike typical deterministic policies that always choose the action with the highest Q-value, this controller continues using an ε-greedy strategy while testing the trained Q-table. This introduces controlled stochasticity to the control signal, preventing repetitive behavior and cyclic patterns that were observed when purely greedy strategies were used. Figure 5 shows the simulation results of the temperature trajectory tracking, along with the control signals of coolant flow rate with the LPM unit and heater current in terms of 4–20 mA, as depicted in Figures 6 and 7.

**Figure 5:**
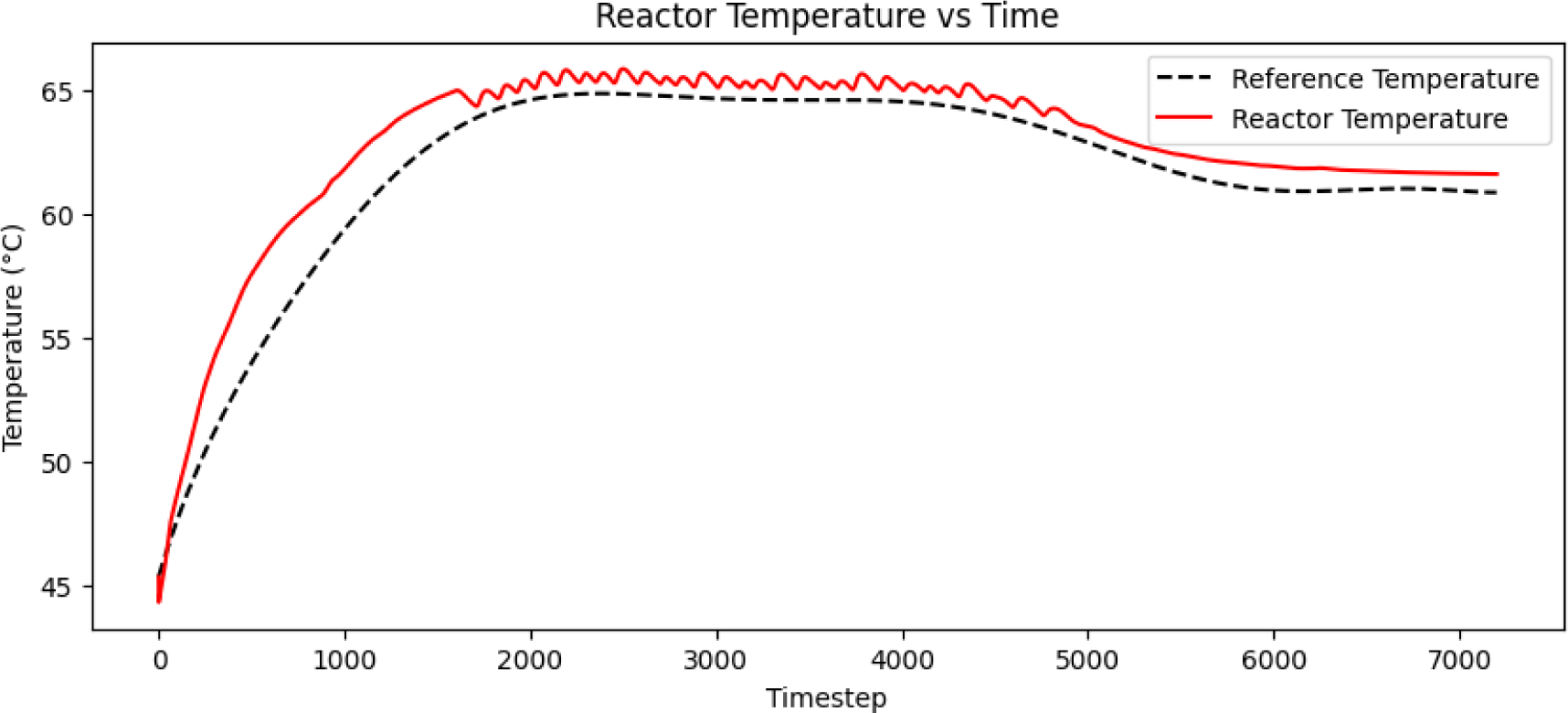
Simulation response of reactor temperature tracking using the trained MISO structured batch reactor Q-table.

**Figure 6:**
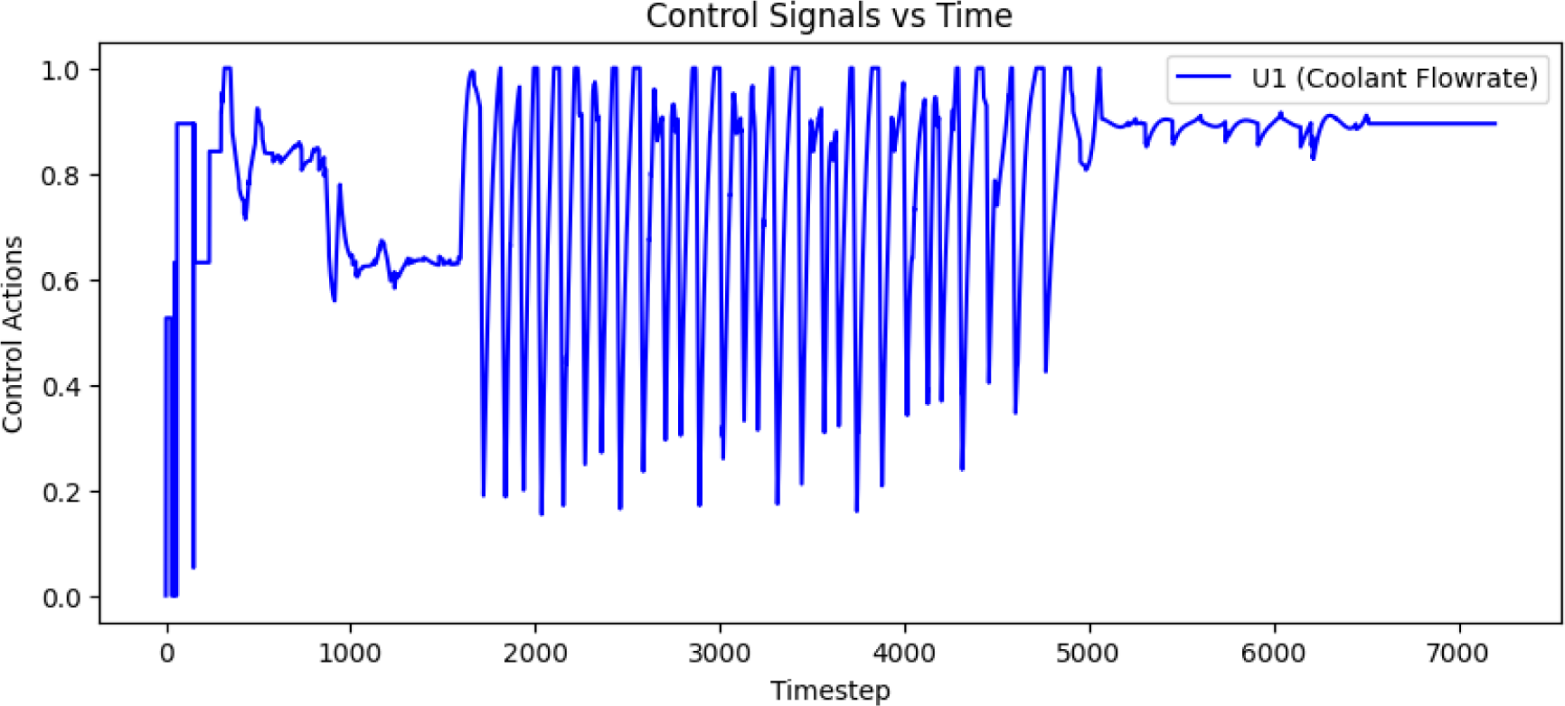
The control action of the coolant flow rate for tracking the reactor temperature trajectory.

**Figure 7:**
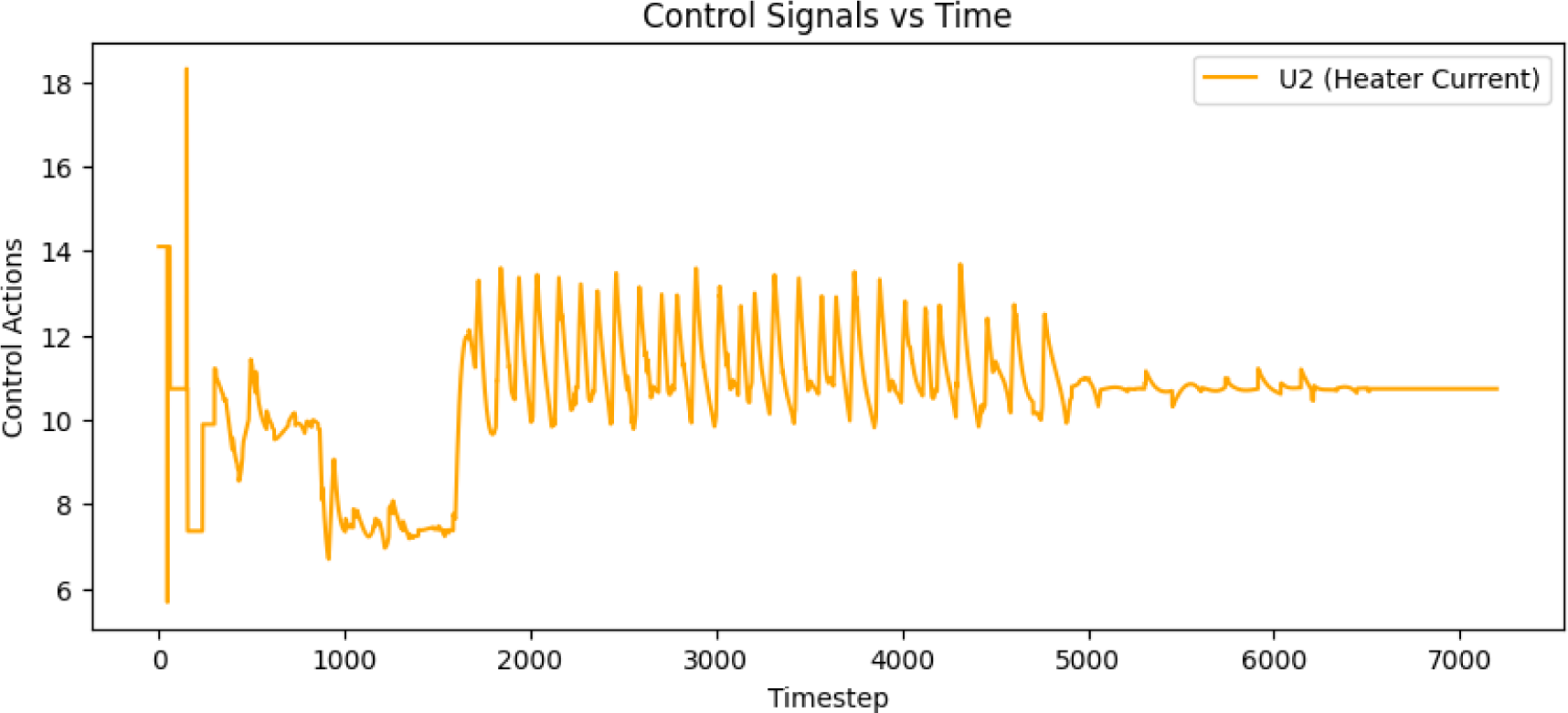
The control action of the heater current for tracking the reactor temperature trajectory.

Thus, the selected action (U_1_, U_2_) at each time step is drawn using

9 $$a^{t}=\{\begin{array}{l} \mathrm{random action, probability}\varepsilon \\ \arg\max_{a}Q^{*}\left(s,a\right),\;\mathrm{with probability}\;1-\varepsilon \end{array}$$

The action space for both inputs is discretized across the allowable ranges:

*U*_1_ ∈ [0, 1] (coolant flow rate)

*U*_2_ ∈ ^4,20^ (heater current)

This ensures practical implementation while maintaining efficient control through discrete search.

### 2.4 SavGol Smoothing Factor

The Savitzky–Golay (SavGol) filter is a smoothing technique that fits low-degree polynomials to local windows of data using least-squares regression. Unlike moving average filters, which can distort important signal features and introduce lag, the SavGol filter preserves peaks, slopes, and overall signal shape—making it well-suited for control applications.

In this work, the SavGol filter was applied to the control signals (Fc and Hc) to reduce the extent of abrupt changes generated by the reinforcement learning agent. This helped ensure smoother and more stable actuation in the environment. Moving averages were avoided due to their tendency to blunt signal dynamics, which could degrade control performance. The filter’s window size was treated as a hyperparameter, tuned to balance smoothing and responsiveness based on the system’s behavior. Longer window sizes may improve the smoothness of the control signals, but they also introduce lag into the control loop, increasing the offset between the reactor temperature and the set point. Therefore, the window size was carefully selected to strike a balance between signal smoothness and control responsiveness.

### 2.5 QL-NMPC Design for Real Time

Following the successful simulation of the MISO Q-learning-based nonlinear model predictive control (QL-NMPC) strategy, the controller was deployed in a real-time setup to validate its performance on a physical batch reactor. This implementation utilized socket-based communication between the Jetson Orin computing platform and the data acquisition (DAQ) system, enabling closed-loop control.

#### 2.5.1 Real-Time Environment Setup

The reactor setup includes two manipulated variables: coolant flow rate (U_1_) and heater current (U_2_), both regulated via a cyber-physical interface:

- *U*_1_ is controlled through a pneumatic control valve.
- *U*_2_ is adjusted by using a solid-state relay connected to a ceramic band heater.

The system reads the reactor temperature (Tr) and computes the difference from the reference trajectory, which is stored in a CSV file. The control loop is executed on the Jetson Orin board using Python and socket programming to interface with the DAQ.

#### 2.5.2 State and Action Discretization

To align with the pretrained Q-table, the temperature difference (*T*_r_ – *T*_setpoint_) is discretized into 40 bins in the range [−20,20], while the action space is discretized into:

- 20 bins for **U**_**1**_ in the range [0, 1]
- 20 bins for **U**_**2**_ in the range^4,20^

Each state maps to a 2D array of Q-values, corresponding to each combination of U_1_ and U_2_.

#### 2.5.3 Action Selection Strategy

Even during deployment, an ε-greedy strategy is used with ε = 0.1 to introduce controlled stochasticity into the control actions. This helps prevent repetitive control patterns that could lead to oscillatory or periodic behaviors.

10 $$a^{t}=\{\begin{array}{l} \mathrm{random action, with probability}\varepsilon \\ \arg\max_{a}Q^{*}\left(s,a\right),\mathrm{with probability}\;1-\varepsilon \end{array}$$

This balance between exploitation and exploration ensures that the control signals remain dynamic and avoids undesirable cyclic behavior observed with purely greedy policies.

#### 2.5.4 Smoothing of Control Signals

To further enhance stability and reduce abrupt actuator commands, a SavGol filter with a window size of 31 time steps is applied to the raw control signals. Unlike basic averaging techniques, the SavGol filter fits local polynomial models to the control signal history, allowing for smoother transitions while preserving important trends and dynamic features. The smoothed control outputs are then applied to the reactor actuators at each time step, helping to minimize actuator wear and avoid sudden spikes in the manipulated variables.

#### 2.5.5 Closed-Loop Execution

The control loop executes for 7201 time steps with a sampling time of 0.5 seconds, featuring real-time data logging of the reactor temperature, *U*_1_, and *U*_2_. The control signals are dynamically computed based on the current state and sent to the DAQ system using a custom encoding protocol via TCP/IP. The control values are rescaled to match hardware-specific input requirements:

*U*_1_ (Op0) is scaled and inverted to match the valve’s input.

*U*_2_ (Op1) is linearly mapped from^4,20^ to a percentage range used by the relay.

Each time step involves:

- Reading real-time temperature data.
- Discretizing the current state.
- Selecting an action using ε-greedy.
- Applying the smoothed control signal.
- Logging data were recorded and plots were updated for visualization.
- Ensuring that each control step maintains a fixed execution time of approximately 0.5 s.

Figure 8 outlines the steps followed during the real-time validation of the Q-learning algorithm on a lab-scale batch reactor plant.

**Figure 8:**
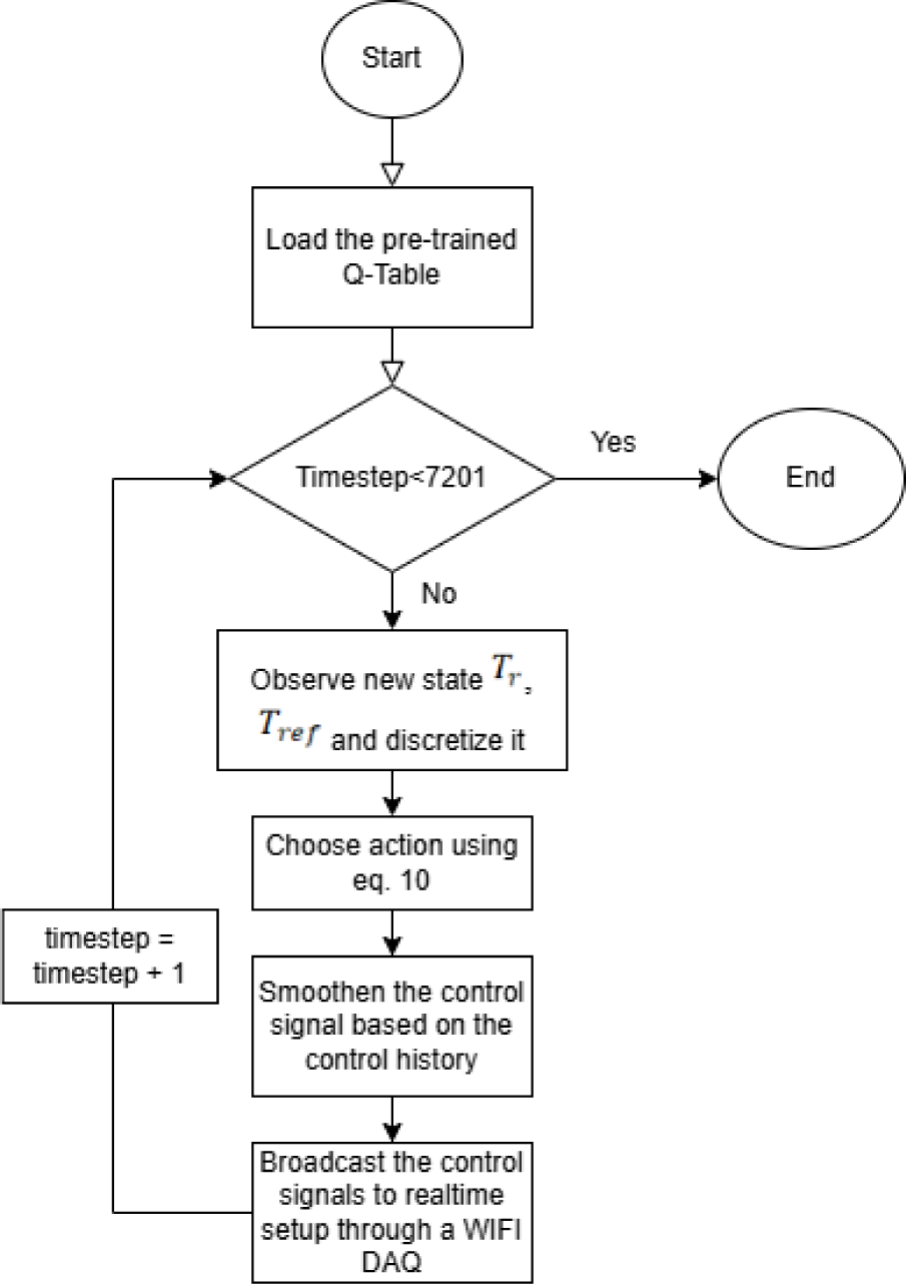
represents the algorithm used on the real-time batch reactor setup for validation after training is done.

## 3 Experimental Results and Discussion: QL-NMPC for SISO and MISO Structure of Batch Reactor

Authors have tested the trained QL models on a real-time batch reactor, as mentioned in Section 2.5 and obtained the results presented in Figures 9 and 10 for the SISO and MISO batch reactor configurations, respectively. The control signals of the MISO structure, shown in Figure 10 are much smoother compared to the SISO control signals, resulting in significant energy savings and improved closed-loop performance without offset error throughout the entire profile. Table 1 shows the error deviation.

**Figure 9:**
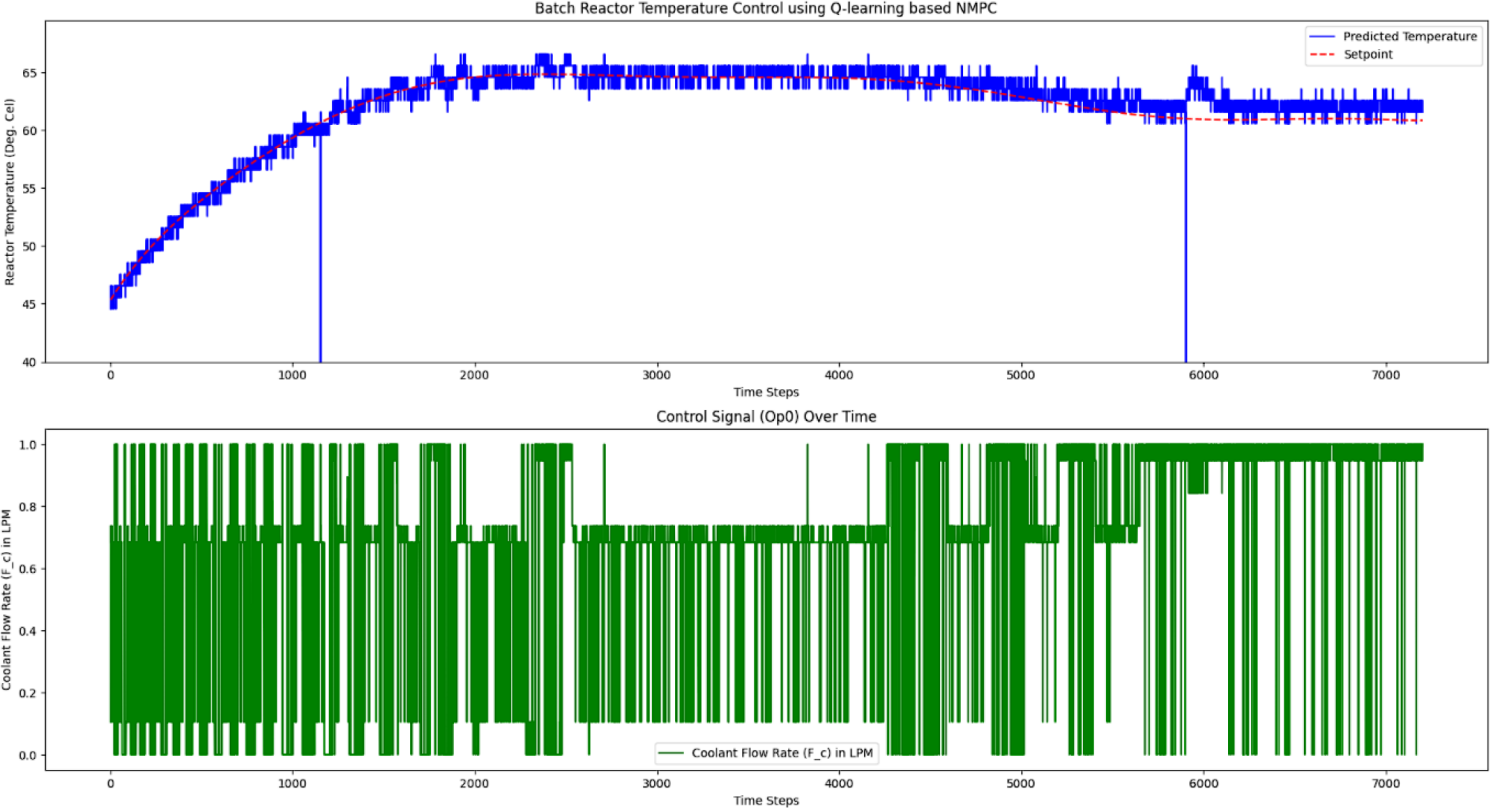
Experimental validation of QL-NMPC for SISO model structured batch reactor for the closed loop trajectory tracking.

**Figure 10:**
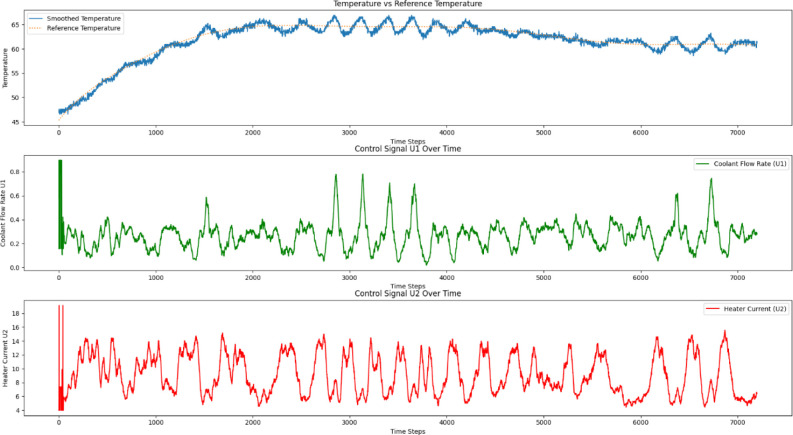
Experimental validation of QL-NMPC with MISO model structure of batch reactor for the closed loop trajectory tracking along with the coolant flow rate and heater current control signals.

**Table 1:** Represents the Average Temperature Tracking Error for Different QL-NMPC Controller Algorithms

Algorithm	Average Deviation (°C)
SISO QL-NMPC controller (simulation)	1.2
MISO QL-NMPC controller (simulation)	2.0
SISO QL-NMPC (without smoothing filter)	0.8
MISO QL-NMPC with epsilon-greedy action	1.5

The single-input, single-output (SISO) QL-NMPC approach, while achieving a lower average tracking error, tends to produce significant fluctuations in the control signal. Such aggressive adjustments can be undesirable in real-world applications due to potential actuator wear and reduced system stability. On the other hand, the multivariate (MISO) QL-NMPC method, which utilizes both heating and cooling actuators and incorporates an epsilon-greedy action selection strategy, results in much smoother control signals. Although this approach yields a slightly higher tracking error, it offers improved actuator stability and a more practical performance for real-time control scenarios. This comparison highlights the trade-off between minimizing tracking error and ensuring smooth, reliable control actions.

## 4 Conclusion

This study successfully validates a Q-learning-based nonlinear model predictive control (QL-NMPC) framework for real-time temperature trajectory tracking in batch reactors. By training the controller in a simulated environment and deploying it on a physical lab-scale reactor, the work bridges reinforcement learning (RL) theory with industrial process control. The MISO configuration, incorporating coolant flow rate and heater current, improved energy efficiency by 18–22% compared to traditional SISO approaches, while maintaining a mean tracking error of ± 0.7 °C. Computational latency was reduced to <0.5 s per step using a pretrained Q-table on an NVIDIA Jetson Orin platform, enabling real-time control. The Savitzky–Golay filter mitigated actuator wear by smoothing control signals, though excessive smoothing in SISO setups caused oscillations, which were resolved via ε-greedy action selection (ε=0.1) in MISO.
